# δ-Tocotrienol, Isolated from Rice Bran, Exerts an Anti-Inflammatory Effect via MAPKs and PPARs Signaling Pathways in Lipopolysaccharide-Stimulated Macrophages

**DOI:** 10.3390/ijms19103022

**Published:** 2018-10-04

**Authors:** Junjun Shen, Tao Yang, Youzhi Xu, Yi Luo, Xinyue Zhong, Limin Shi, Tao Hu, Tianyi Guo, Ying Nie, Feijun Luo, Qinlu Lin

**Affiliations:** 1 Hunan Key Laboratory of Grain-Oil Deep Process and Quality Control, Hunan Key Laboratory of Processed Food for Special Medical Purpose, College of Food Science and Engineering, National Engineering Laboratory for Deep Process of Rice and Byproducts, Central South University of Forestry and Technology, Changsha 410004, Hunan, China; shenjunjun@yeah.net (J.S.); yangtao807@163.com (T.Y.); xuyouzhi123@hotmail.com (Y.X.); zhongxinyue111@163.com (X.Z.); Shilm6666@126.com (L.S.); hutao0829@hotmail.com (T.H.); guotianyib11@hotmail.com (T.G.); ny198722@hotmail.com (Y.N.); 2 Department of Clinic Medicine, Xiangya School of Medicine, Central South University, Changsha 410008, Hunan, China; YiLuo603@hotmail.com

**Keywords:** rice bran, δ-tocotrienol, inflammation, MAPKs, PPARs, RAW264.7

## Abstract

δ-Tocotrienol, an important component of vitamin E, has been reported to possess some physiological functions, such as anticancer and anti-inflammation, however their molecular mechanisms are not clear. In this study, δ-tocotrienol was isolated and purified from rice bran. The anti-inflammatory effect and mechanism of δ-tocotrienol against lipopolysaccharides (LPS) activated pro-inflammatory mediator expressions in RAW264.7 cells were investigated. Results showed that δ-tocotrienol significantly inhibited LPS-stimulated nitric oxide (NO) and proinflammatory cytokine (TNF-α, IFN-γ, IL-1β and IL-6) production and blocked the phosphorylation of c-Jun N-terminal kinase (JNK) and extracellular regulated protein kinases 1/2 (ERK1/2). δ-Tocotrienol repressed the transcriptional activations and translocations of nuclear factor-kappa B (NF-κB) and activator protein-1 (AP-1), which were closely related with downregulated cytokine expressions. Meanwhile, δ-tocotrienol also affected the PPAR signal pathway and exerted an anti-inflammatory effect. Taken together, our data showed that δ-tocotrienol inhibited inflammation via mitogen-activated protein kinase (MAPK) and peroxisome proliferator-activated receptor (PPAR) signalings in LPS-stimulated macrophages.

## 1. Introduction

Inflammation is a significant mechanism of the immune pathogenesis and against different harmful stimuli [1]. Inflammation may result in tissue injury, infection and stress or exposure to bacterial components, such as lipopolysaccharide (LPS) [2,3]. LPS is a component of the cell wall of gram-negative bacteria and was used to induce the cell inflammation model in macrophage RAW264.7. During inflammatory response, macrophages play an important role to provide a defense against the foreign stimuli. Macrophages take part in the inflammatory process through regulating a series of inflammatory cytokines, such as tumor necrosis factor-α (TNF-α), interferon-γ (INF-γ), interleukin-1β (IL-1β), IL-6 and IL-8 [4]. On the other hand, macrophages also stimulate the expressions and secretions of inflammatory mediators, including nitric oxide (NO) and prostaglandin E2 (PGE2). NO release is controlled by inducible NO synthase (iNOS) and PGE2 secretion is synthesized by cyclooxygenase-2 (COX-2) [5]. Although the cellular signaling pathways and molecular mechanisms of the inflammation response are very complicated, mitogen-activated protein kinase (MAPK) is an important pathway in the initiation and development of the inflammation process. MAPKs are the specific protein family of serine/threonine kinases, which can transmit signal by sequential phosphorylation events. Meanwhile, lipids play essential roles in almost all inflammation processes [6,7]. Peroxisome proliferator-activated receptors (PPARs) are key important transcript factors which are involved in lipid metabolism and inflammation, and PPAR pathway is closely related with developing chronic inflammation, diabetes, obesity, hypertension and hyperlipidemia [8,9,10,11]. PPAR super family contains α-, γ-, δ-, (PPARα, PPARγ and PPARδ), which are ligand-regulated transcription factors and belong to the nuclear hormone receptors [7]. Specific ligands like unsaturated fatty acids participate in the regulation of physical metabolic pathways. Interestingly, δ-tocotrienol is an unsaturated fatty acid [12,13]. Although PPARs have many similarities among each different isoform, they have their own particular functions, tissue distributions, biomedical properties and unique reactions to different ligands [14,15]. PPAR signaling can also activate activator protein-1 (AP-1) and activate mitogen-activated protein kinase (MAPK) signaling, which forms a complex signal pathway net via cross-talking different pathways in the response. However, the complex mechanism of inflammation is still not clear [15,16].

δ-Tocotrienol is a bioactive component of rice, which is a common staple food consumed worldwide [17,18]. Since rice is the most important crop around the world harvested from over 100 countries, its components including rice starch, δ-tocotrienol and other vitamins deserve more attention [19]. Rice bran is an important by-product of rice acquired from rice milling process. Rice bran and its components exert multiple biological effects [17,18]. δ-Tocotrienol is a member of vitamin E family which can be extracted from rice bran. δ-Tocotrienol can also obtained from some plant resources such as palm, coconut and grains like oat, wheat, maize and rye [20,21,22]. Vitamin E compounds family has members named α-, β-, γ-, δ- tocopherols and α-, β-, γ-, δ-tocotrienol, all of the eight chemically distinct isomers constitute vitamin E [23,24,25]. Unlike saturated tocopherols, tocotrienols are unsaturated forms of vitamin E and own an isoprenoid side chain. δ-Tocotrienol was found to take part in a lot of health-promoting functions which include anti-diabetic, cholesterol-lowering, anticancer, antihyperlipidemic, immunomodulatory effects, antioxidant and anti-inflammation, but its molecular mechanism is not clear [26].

In this study, we used an LPS-induced macrophage inflammation model to evaluate the anti-inflammation function of δ-tocotrienol and explore if δ-tocotrienol inhibits inflammation through MAPK and PPAR pathways. Moreover, we investigated the cross-talk of MAPK and PPAR pathways and how δ-tocotrienol prevented inflammation in the in vitro model. 

## 2. Results 

### 2.1. Isolation and Purification of δ-Tocotrienol from Rice Bran 

**Figure 1:**
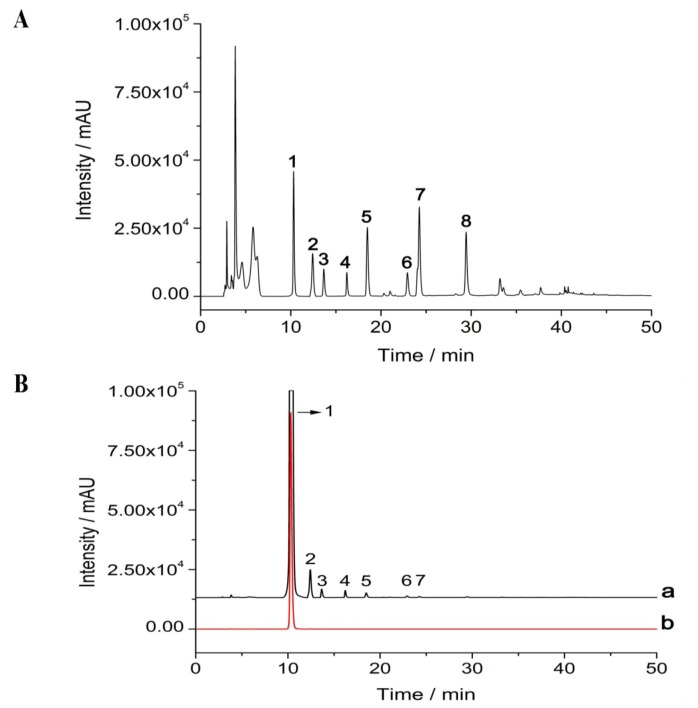
Isolation and purification of δ-tocotrienol from rice bran. (**A**) HPLC analyzed the different peaks of oil from the rice bran. (**B**) HPLC identified the purified δ-tocotrienol. The red color peak is the standard sample of δ-tocotrienol. HPLC: high performance liquid chromatography; “a”: experiment sample; “b”: standard sample. Peak 1: δ-tocotrienol; peak 2: β-tocotrienol; peak 3: γ-tocotrienol; peak 4: α-tocotrienol; peak 5: δ-tocopherol; peak 6: β-tocopherol; peak 7: γ-tocopherol; peak 8: α-tocopherol.

Crude rice bran oil was extracted from rice bran by supercritical carbon dioxide extraction. The rice bran oil extracts from rice bran were then identified by high performance liquid chromatography (HPLC). As Figure 1A indicates, there are 8 peaks in rice bran extracts which include α-tocopherol, β-tocopherol, γ-tocopherol, δ-tocopherol, α-tocotrienol, β-tocotrienol, γ-tocotrienol and δ-tocotrienol. The yield of δ-tocotrienol that was isolated from rice bran oil that was prepared by carbon dioxide extraction was 132.7 μg/kg. After the purification of δ-tocotrienol from the rice bran oil using the Shim-pack PREP-ODS (20.0 mm × 250 mm, 15 µm, Shimadzu Co., Ltd., Kyoto, Japan), δ-tocotrienol and related substance, which was extracted from rice bran, was finally detected by the fluorescence detector (Prominence RF-20A/Axs, Shimadzu Co., Ltd., Kyoto, Japan). The purity of δ-tocotrienol that was extracted from rice bran is 96.2% (Figure 1B).

### 2.2. The Toxicity of δ-Tocotrienol on RAW264.7 Cells 

**Figure 2:**
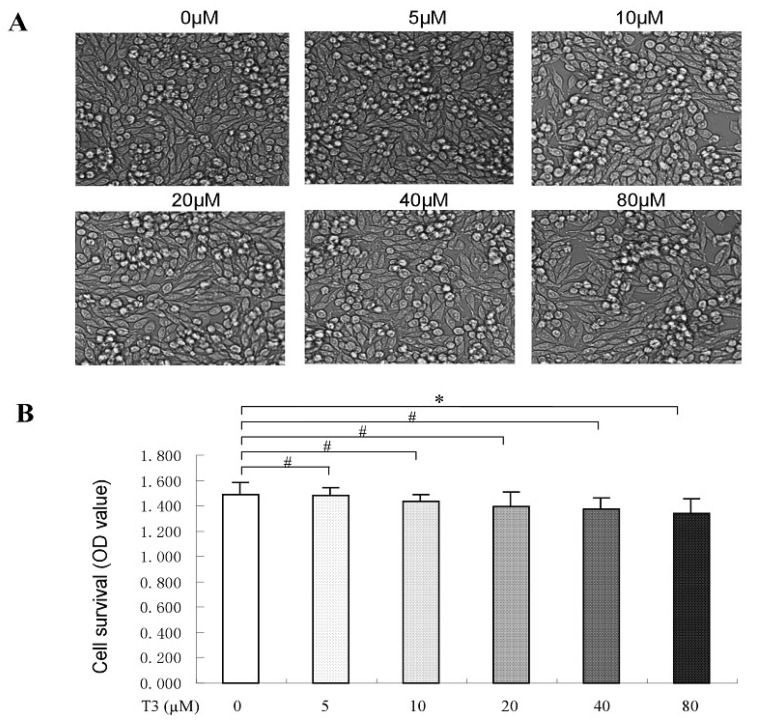
Effect of δ-tocotrienols on the phenotype of LPS-induced RAW264.7. (**A**) The morphologic change of LPS-induced RAW264.7 after treatment of δ-tocotrienols; (**B**) Effects of δ-tocotrienols in cell viability. LPS: liposaccharide; OD: optical density; T3: δ-tocotrienol. Data are expressed as the mean ± SD of three independent experiments. *: *p* < 0.05; #: *p* > 0.05.

To investigate the cell toxicity of δ-tocotrienol, different concentrations of δ-tocotrienol were divided into 6 groups: control, δ-tocotrienol (5 μM), δ-tocotrienol (10 μM), δ-tocotrienol (20 μM), δ-tocotrienol (40 μM), δ-tocotrienol (80 μM). The phenotype of the cells after δ-tocotrienol treatment was observed by optical microscope (Leica, Solms, Germany). The optical density (OD) value, which stands for the rate of cell survival from control group to δ-tocotrienol (80 μM), had no significant differences (*p* > 0.05). Results revealed that under each concentration of δ-tocotrienol treatment, this alone had no effect on murine macrophages RAW264.7. The cell viability of different intensities of δ-tocotrienol on RAW264.7 cells was determined by trypan blue dye exclusion or a quantitative colorimetric assay with MTS [3-(4,5-diethylthiazol-2-yl)-5-(3-carboxymethoxyphenyl)-2-(4-sulfo phenyl)-2H-etrazolium,inner salt] assay. The data showed that δ-tocotrienol exerted no obvious suppression effect on cell viability of RAW264.7 (0–40 μM), see Figure 2A,B. Although our data showed that 40 μM δ-tocotrienol did not affect cell viability, we selected 20 μM δ-tocotrienol in the next experiments to avoid any cytotoxicity of δ-tocotrienol. 

### 2.3. δ-Tocotrienol Downregulated the Expressions of Proinflammatory Factors

**Figure 3:**
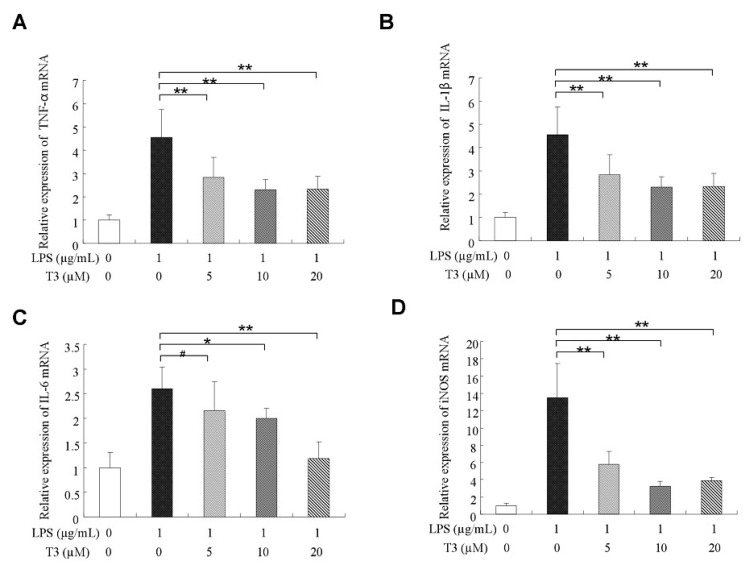
δ-Tocotrienols inhibit mRNA expression of inflammatory cytokines/mediator in LPS-stimulated RAW264.7 cells. (**A**) Relative expression of TNF-α mRNA; (**B**) Relative expression of IL-1β mRNA; (**C**) Relative expression of IL-6 mRNA; (**D**) Relative expression of inducible nitric oxide synthase (iNOS) mRNA. T3: δ-tocotrienol; LPS: lipopolysaccharide. The data came from three independent experiments. Comparing with LPS group, *: *p* < 0.05; **: *p* < 0.01; #: *p* > 0.05.

**Figure 4:**
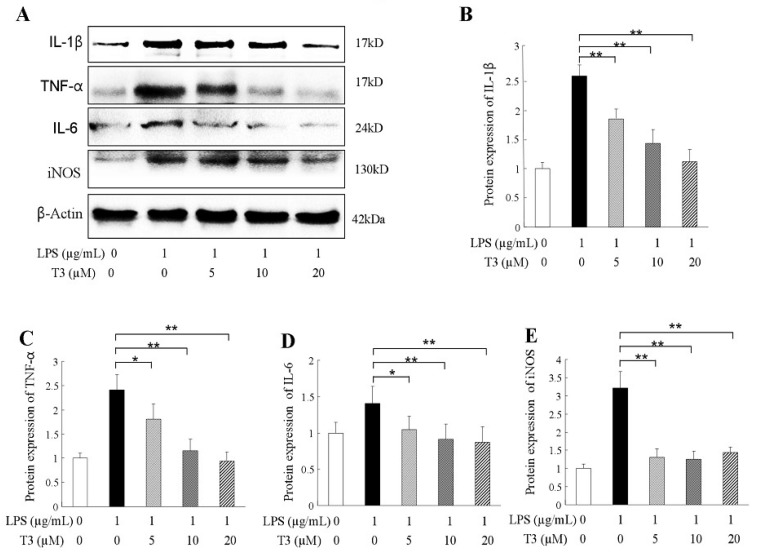
δ-Tocotrienols inhibit protein expression of inflammatory factors in LPS-activated RAW264.7 cells. (**A**) Representative image of Western blotting from 3 independent experiments; (**B**) Protein expression of IL-1β; (**C**) Protein expression of TNF-α; (**D**) Protein expression of IL-6; (**E**) Relative expression of iNOS mRNA. iNOS: inducible nitric oxide synthase; LPS: lipopolysaccharide T3: δ-tocotrienol. Comparing with LPS group, *: *p* < 0.05; **: *p* < 0.01.

During suffering inflammatory disease, the proinflammatory factors, such as TNF-α, IL-1β, IL-6, and mediators of iNOS were always overproduced [27]. In our study, the mRNA expressions of proinflammatory factors were assessed by real-time quantitative PCR (RT-qPCR). The results indicated that the upregulation expressions of pro-inflammatory cytokine mRNAs in the LPS-stimulated group were significantly higher than that of the control group (Figure 3A–D). δ-tocotrienol treatments decreased the mRNA expressions of TNF-α, IL-1β, IL-6 and iNOS in a dosage-dependant manner, as shown in Figure 3A–D. Western blotting further confirmed that δ-tocotrienol treatments (5 μM, 10 μM, 20 μM) inhibited the protein expression levels of TNF-α, IL-1β, IL-6 and iNOS in the LPS-stimulated macrophage (Figure 4A–E). Taken together, δ-tocotrienol exerts an anti-inflammatory effect via decreasing expressions of inflammatory factors. 

### 2.4. Effect of δ-Tocotrienol on MAPKs in LPS-Stimulated RAW264.7 Cells

**Figure 5:**
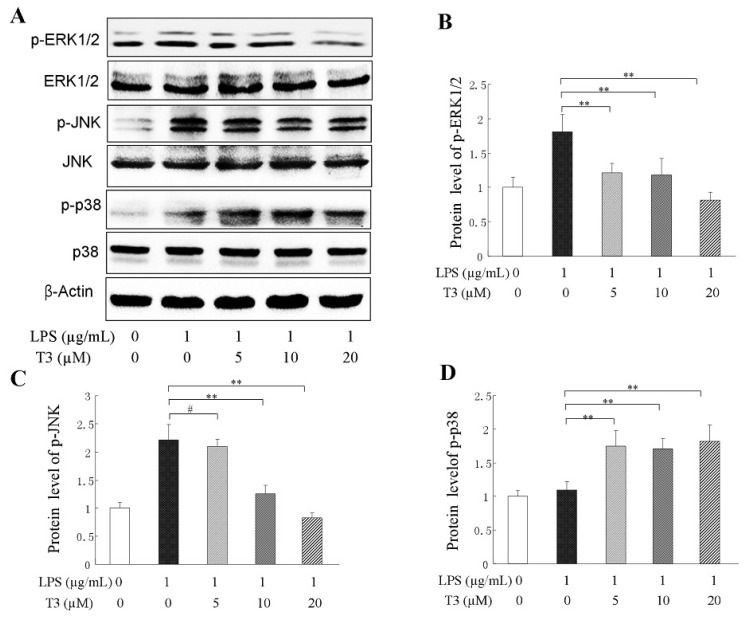
δ-Tocotrienol prevents MAPK pathways in LPS-stimulated RAW264.7 cells. (**A**) Representative image of Western blotting from 3 independent experiments; (**B**) Protein level of p-ERK1/2; (**C**) Protein level of p-JNK; (**D**) Protein level of p-p38. ERK1/2: extracellular regulated protein kinases; JNK: c-Jun N-terminal kinase; LPS: lipopolysaccharide; MAPK: mitogen-activated protein kinase; T3: δ-tocotrienol. The values represent the means ± SD. Comparing with LPS group, **: *p* < 0.01; #: *p* > 0.05.

It has been approved that mitogen-activated protein kinases (MAPKs) are phosphorylated in the inflammatory response, and that the MAPK transduction pathway activation is the key to signaling to regulate the expression of inflammatory cytokine [28,29]. To explore the anti-inflammatory mechanism of δ-tocotrienol, the phosphorylation situation of 3 subtypes of MAPKs, including c-Jun N-terminal kinase (JNK), extracellular signal-regulated kinase 1/2 (ERK1/2), and p38 were analyzed by Western blotting in the LPS-stimulated RAW264.7 cells. As shown in Figure 5A, LPS treatment caused an obvious increase of phosphorylation of ERK1/2, JNK and p38; Adding δ-tocotrienol resulted in a reduction of phosphorylation of ERK1/2, JNK (see Figure 5B,C), however δ-tocotrienol did not inhibit the phosphorylation of p38 (see Figure 5D) in a dosage-dependent manner. Our data showed that δ-tocotrienol that was treated with 20 μM was the effective dosage for the inhibition of ERK1/2 and JNK phosphorylation, and the protein contents of p-ERK1/2 and p-JNK were reduced to 76.6% and 28.9% (Figure 5B,C), respectively. These results suggest that δ-tocotrienol exerts an anti-inflammatory effect which may be mediated by inhibiting the ERK/JNK activation (phosphorylation) in the LPS-stimulated cell inflammation model.

### 2.5. Effect of δ-Tocotrienol on NF-κB /AP-1 Activities and Translocations 

**Figure 6:**
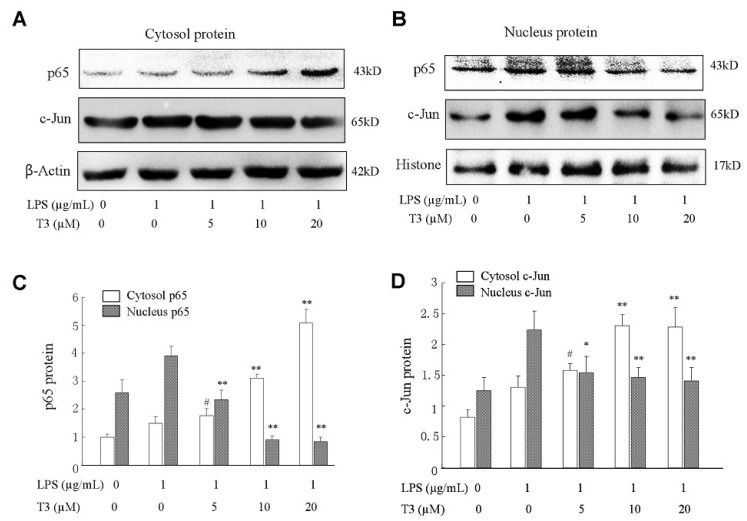
δ-Tocotrienol inhibits the nuclear translocations of NF-κB and AP-1. (**A** and **C**) Effect of δ-tocotrienol on the protein content of p65 and c-Jun by Western blotting analysis in cytosol; (**B** and **D**) Effect of δ-tocotrienol on the protein content of p65 and c-Jun by Western blotting analysis in nucleus. AP-1: activator protein-1; LPS: lipopolysaccharide; NF-κB: nuclear factor-kappa B; T3: δ-tocotrienol. The figure shown here are representative data from three independent experiments. The values represent the means ± SD. Comparing with LPS group, *: *p* < 0.05; **: *p* < 0.01; #: *p* > 0.05.

**Figure 7:**
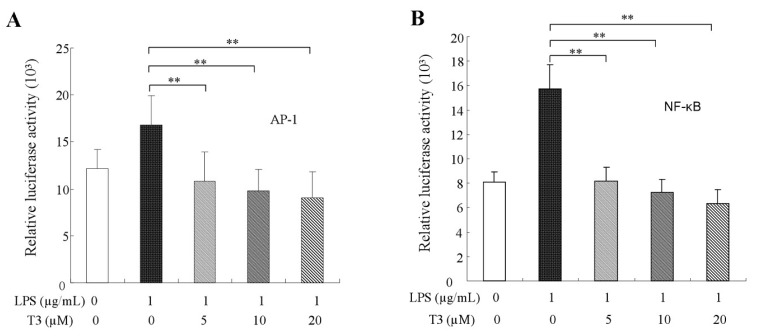
δ-Tocotrienol inhibits the transcriptional activities of NF-κB and AP-1. (**A**) Effect of δ-tocotrienol on the transcriptional activity of NF-κB in LPS-stimulated RAW264.7 cells; (**B**) Effect of δ-tocotrienol on the transcriptional activity of AP-1 in LPS-stimulated RAW264.7 cells. T3: δ-tocotrienol; LPS: lipopolysaccharide. The values represent the means ± SD. Comparing with LPS group, **: *p* < 0.01.

Transcription factors AP-1 and NF-κB are involved in many biological functions. In the inflammatory response, inflammatory cytokine, such as TNF-α, IFN-γ, IL-1β, IL-6 and iNOS have several AP-1 and NF-κB binding sites and the two transcript factors can directly bind the promoter of those genes to regulate inflammatory cytokine expressions [30,31]. After exposure to LPS alone, our data showed that the contents of p65 and c-Jun in the nuclei were increased significantly and LPS promoted the nuclear translocation of p65 and c-Jun (Figure 6A,B). After treatments of different dosages of δ-tocotrienol, the contents of p65 and c-Jun in the nuclei were obviously reduced in a dose dependent manner compared with the LPS group and, in contrast, the contents of p65 and c-Jun in the cytoplasm were increased in RAW264.7 cells. Compared with the LPS group, the relative luciferase activities of NF-κB were reduced to 57.9%, 43.6% and 43.5% by reporter gene analysis (Figure 6A,C), and the relative luciferase activities of AP-1 were reduced to 63.2%, 53.8% and 41.4% (Figure 6B,D), respectively. This suggests that δ-tocotrienol may reduce inflammatory cytokine expressions via inhibiting NF-κB and AP-1 activation in the LPS-induced RAW264.7 cells. Furthermore, pNF-κB-Luc and pAP-1-Luc reporter genes were used to detect the effect of δ-tocotrienol on the transcriptional activities of NF-κB and AP-1. Consistent with the nuclear translocation data, luciferase reporter assays found that δ-tocotrienol significantly inhibited NF-κB and AP-1 activities in a dose-dependent manner, as shown in Figure 7A,B. MAPKs are found to be close with AP-1 and NF-κB activations [29,32]. Our results suggest that δ-tocotrienol may affect inflammatory cytokine expressions via MAPK/NF-κB and MAPK/AP-1 pathways. 

### 2.6. Effect of δ-Tocotrienol on the Activities of PPARα and PPARγ In Vitro Models 

**Figure 8:**
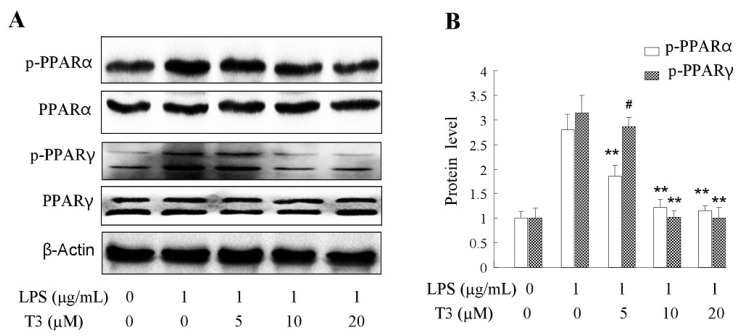
δ-Tocotrienols inhibit PPAR signaling in LPS-stimulated RAW264.7 cells. (**A**) Representative image of PPARs Western blotting from 3 independent experiments; (**B**) Protein level of p-PPARs. LPS: lipopolysaccharide; PPAR: peroxisome proliferator-activated receptor; T3: δ-tocotrienol. Comparing with LPS group, **: *p* < 0.01; #: *p* > 0.05.

Peroxisome proliferator-activated receptors (PPARs) are ligand-activated nuclear receptors which have three isoforms: PPARα, PPARβ/δ, PPARγ, and each isoform has its own physiological functions. PPARα was supposed to be involved in heart failure [33,34]. Both PPARα and PPARβ/δ have overlapping functions in cardiovascular diseases [34]. PPARγ is well known for its therapeutic potency of metabolic syndrome, type 2 diabetes and obesity [12,35,36]. In this study, we estimated the effect of δ-tocotrienol on the PPAR pathway in the LPS-stimulated RAW264.7 cells. The results revealed that in LPS-induced RAW264.7, δ-tocotrienol significantly inhibited the phosphorylation of PPARα at concentration of 5 μM, 10 μM, 20 μM in a dosage-dependent manner compared with the control group, as shown in Figure 8A,B. In this study, our data also demonstrated that δ-tocotrienol significantly depressed the phosphorylation of PPARγ at concentrations of 5 μM, 10 μM, 20 μM in a dosage-dependent manner compared with the control group.

### 2.7. Effect of δ-Tocotrienol on MAPKs and PPARs Signaling Models 

**Figure 9:**
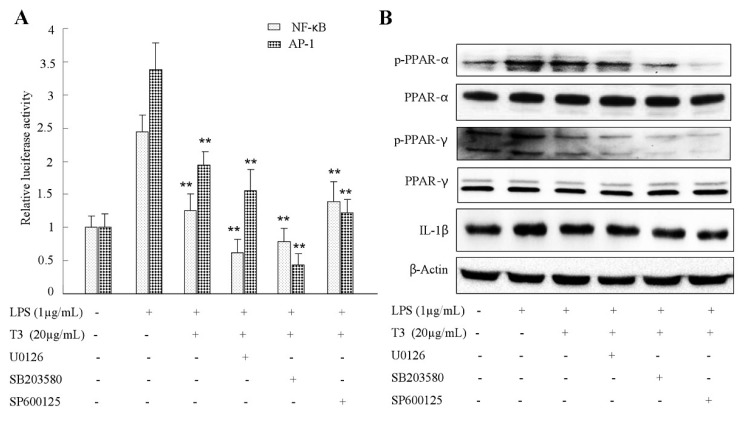
δ-Tocotrienols inhibit MAPK/AP-1/NFκB and MAPK-PPAR signalings in LPS-stimulated RAW264.7 cells. (**A**) RAW264.7 cells were pretreated with the p38MAPK inhibitor SB203580 with 20 μM, the JNK inhibitor SP600125 with 20 μM, and the ERK1/2 inhibitor U0126 with 10 μM for 30 min, and were then treated with/without δ-tocotrienols for 2 h, and finally treated with LPS for 12 h. The results shown here are representative data from three independent experiments. (**B**) The phosphorylated or total forms of PPARs after treatment of MAPK inhibitors were measured by Western blotting. T3: δ-tocotrienol; LPS: lipopolysaccharide.

To investigate whether the MAPK signaling pathway interacts with PPARs, which take part in the anti-inflammation of δ-tocotrienol, LPS-induced RAW264.7 cells were treated with the p38MAPK inhibitor SB203580, the JNK inhibitor SP600125 and the ERK1/2 inhibitor U0126. As shown in Figure 9A, in contrast to the δ-tocotrienol treated group, SB600125, U0126 and SP203580 treatment all suppressed the phosphorylation of PPARγ (Ser112) protein which was stimulated by LPS in the presence of δ-tocotrienol. Moreover, compared with the δ-tocotrienol treated group (Figure 9B), both SP600125 and U0126 treatment inhibited the phosphorylated of PPARα (Ser384), while SB203580 treatment did not activate the phosphorylation of PPARα (Ser384) level. These data indicated that δ-tocotrienol inhibited PPARα activation via inhibiting JNK and ERK1/2 activities; δ-tocotrienol inhibited PPARγ phosphorylation through inhibiting p38, JNK and ERK1/2 activities.

## 3. Discussion

In the present study, we demonstrated the anti-inflammatory effects and molecular mechanisms of δ-tocotrienol through the MAPKs/AP-1 and PPARs/AP-1 pathways. In previous studies, it is reported that δ-tocotrienol depressed the expressions of proinflammatory genes, however the role of δ-tocotrienol in the MAPK/AP-1 and PPARs/AP-1 pathways and the interactions between these two pathways remains unclear [15]. Our results verified that δ-tocotrienol significantly depressed the productions of IL-1β, IL-6, iNOS and TNF-α, meanwhile it does not have cytotoxicity on LPS-induced RAW264.7, as confirmed by MTS assay.

Rice bran oil and rice germ oil, together with palm oil have been used traditionally as cooking oil which have a high content of tocotrienol [37]. Recent studies showed that vitamin E components, like tocotrienol-rich fraction, had already been used as dietary complements to prevent breast cancer and hypercholesterolemia and its anti-inflammatory activity is the greatest compared with that of a-tocopherol and a-tocopheryl acetate [38,39,40]. Vitamin E components, such as γ-tocopherol, δ-tocopherol and γ-tocotrienol, have specific anti-inflammatory and antioxidant effects which are superior to those of α-tocopherol [41]. Several studies have shown that γ-tocotrienol inhibited LPS-stimulated RAW264.7 macrophages and IL-1β-activated lung epithelial cells through NF-κB and JAK-STAT6 or JAK-STAT3 signaling pathways [42,43]. In human endothelial cells, δ-tocotrienol is the most potent isomer of tocotrienols in depressing the expression of IL-6, ICAM-1, VCAM-1 and NF-κB compared with that of α-, β-, γ-tocotrienol [35]. δ-Tocotrienol has several potential health benefits, such as prevention of certain types of cancer [44], heart diseases and other acute or chronic inflammations. Among the isoforms of tocotrienols, the antioxidant and anti-inflammation functions of γ-tocotrienol were well studied, and only few experiments showed that δ-tocotrienol can decrease the expression of inflammatory factors in macrophages, however its molecular mechanism is unknown.

In the present study, we demonstrated the anti-inflammatory effects and molecular mechanisms of δ-tocotrienol through the MAPKs/AP-1 and PPARs/AP-1 pathways. In previous studies, it is reported that δ-tocotrienol depressed the expressions of proinflammatory genes, however the role of δ-tocotrienol in the MAPK/AP-1 and PPARs/AP-1 pathways and the interactions between these two pathways remains unclear [15]. Our results verified that δ-tocotrienol significantly depressed the productions of IL-1β, IL-6, iNOS and TNF-α, meanwhile it does not have cytotoxicity on LPS-induced RAW264.7, as confirmed by MTS assay.

MAPKs signaling pathway and PPARs signaling pathway were supposed to take part in the occurrence of inflammation. Although the cellular signaling pathways and molecular mechanisms of inflammation activation are very complicated, mitogen-activated protein kinase (MAPK) is a key signaling pathway in the initiation and development of the inflammation process. MAPK can transmit the extracellular information into cytoplasm and nucleus in the end. These serine/threonine kinases include extracellular signal-regulated kinase 1/2 (ERK1/2), extracellular signal-regulated kinase 5 (ERK5) c-Jun NH2-terminal kinase (JNK) and p38 and finally to the NF-кB and AP-1 in cell nucleus. Among PPAR receptors, PPARα was the first to be identified and expressed mainly in liver, heart, kidney and adipose tissues [15,45]. PPARα upregulates the expression of IκB, which inhibits the activation and nuclear translocation of the proinflammatory transcription factor NF-κB [46]. Both PPARα and PPARγ are reported to reduce the NF-κB transcriptional activity [13]. Fatty acids and their derivatives can activate peroxisome proliferator-activated receptors (PPARs) which regulate the expression of signaling pathways genes that are involved in adipogenesis, lipid metabolism, inflammation, type 2 diabetes and the maintenance of metabolic homeostasis [47]. In this study, δ-tocotrienol was found to decrease the production of inflammatory cytokines, such as IL-6, IL-1β and TNF-α with iNOS. Meanwhile in PPARα knock-out young (4-week-old) and senescent mice (42-week old), it was reported that δ-tocotrienol can also decrease the mRNA expressions of IL-6, IL-1β and TNF-α in vivo. However, the mediation of δ-tocotrienol via PPARs and MAPKs signaling is still unclear. In our present study, we found that δ-tocotrienol inhibits inflammation by activation of both PPARα and PPARγ receptors. Indeed, overweight and obesity inflammation are related to the interaction of nutrition, the immune system and metabolic organs [48,49,50]. In low grade chronic inflammation, where the innate immune system and arteries, heart, and brain are involved, all the three PPAR isotypes showed anti-inflammatory effects [31,45]. Both PPARα and PPARγ are reported to reduce the NF-κB transcriptional activity [13]. 

In our present study, we found that δ-tocotrienol inhibited MAPKs activation and downregulated the expression of inflammatory cytokines as IL-1β, IL-6, TNF-α and iNOS in the LPS-stimulated cell inflammation model. For all of those cytokines, they have AP-1 and NF-κB binding sites in the promoters of those genes. c-Jun is a direct target of JNK, and JNK activation will result in AP-1 activation, which promotes the expressions of proinflammatory factors. Our study also confirmed that δ-tocotrienol can inhibit the phosphorylation of JNK and transcriptional activity of AP-1. This means that δ-tocotrienol can inhibit expressions of proinflammatory factors via downregulating JNK (MAPK). δ-tocotrienol inhibited activation ERK1/2 and transcriptional activity of NF-κB, and δ-tocotrienol decreased expressions of proinflammatory factors through ERK1/2/NF-κB, which belonged to another important pathway. The results suggest that both JNK (MAPK) and ERK1/2/NF-κB were involved in the inhibition of the activation of MAPKs. Meanwhile, the effect of δ-tocotrienol on PPARs signaling in LPS activated RAW264.7 cells was estimated by Western blotting. The results demonstrated that the treatment of δ-tocotrienol suppressed the phosphorylation of PPARα and PPARγ and the two different isotypes in LPS that induced murine macrophages in a dosage dependent way. It was found that PPARα and PPARγ can repress the inflammatory response by blocking the activation of NF-κB [51,52]. NF-κB was formed by p65 and p50 proteins, and the phosphorylation of translocation of NF-κB from cytoplasm to nucleus leads to overexpressions of proinflammatory factors [53]. Moreover, when MAPK inhibitors were added to δ-tocotrienol treated RAW264.7 cells, the phosphorylation of PPARγ was further downregulated. On the other hand, both JNK and ERK1/2 inhibitors treatment blocked the phosphorylation of PPARα (Ser384), while p38 inhibitor treated had a weak effect on the phosphorylation of PPARα (Ser384) level. The results indicated that δ-tocotrienol upregulated the phosphorylation of PPARα through JNK and ERK1/2 and upregulated the phosphorylation of PPARγ by JNK and ERK1/2.

In conclusion, it is well known that MAPKs signaling play a key role in inflammation; we demonstrated that δ-tocotrienol repressed the inflammatory response via the inhibition of MAPK/ERK/JNK activation. PPARs are always the target of many drugs which are therapy to metabolic syndrome, dyslipidemia, insulin resistance, hypertension, type 2 diabetes and cardiovascular diseases. This is the first study to report the fact that δ-tocotrienol reduces AP-1 activation during LPS-stimulated inflammatory response, and δ-tocotrienol inhibits inflammatory cytokine expressions via MAPK and PPARs signalings. Further investigation found that crosstalk exists between MAPKs and PPARs, which is involved in the anti-inflammatory effect of δ-tocotrienol. δ-tocotrienol can be developed as a food supplement for diseases like obesity, cardiovascular diseases, diabetes, hypertension and hyperlipidemia, which are closely related to inflammation and chronic low-grade inflammation which MAPKs and PPARs signaling pathways are involved in.

## 4. Materials and Methods

### 4.1. Materials and Reagents 

The rice bran was purchased from Hunan Jinjian Cereals Industry Co., Ltd. (Changde, China). Lipopolysaccharide (LPS) from *Escherichia coli* O127:B8 was obtained from Sigma-Aldrich (St. Louis, MO, USA). Stock solutions of δ-tocotrienol were purchased from Chromadex, Inc. (Irvine, CA, USA), purity 99.4%. Stock solutions of δ-tocotrienol were dissolved in ethanol and were blended by ultrasonic concussion for 5 min. Fetal bovine serum (FBS) and Roswell Park Memorial Institute (RPMI) medium were bought from Gibco (Grand Island, NY, USA). The Nuclear and Cytoplasmic Protein Extraction Kit (P0028), Enhanced BCA protein kit (P0009) and Penicillin and streptomycin were purchased from Beyotime Biotechnology Company (Shanghai, China). Antibody to histone H3 was obtained from Beyotime Biotechnology Company (Nantong, China). Polyclonal antibody against β-Actin (Cat#12620), iNOS (Cat#13120), IL-1β (Cat#12507), IL-6 (Cat#12912), TNF-α (Cat#11948), c-Jun, Phospho-c-Jun (Ser73), ERK1/2, phospho-ERK1/2 (T202/Y204), JNK phospho-JNK (T183/Y185), p38, phospho-p38 (Thr180/Tyr182), PPAR-α, phosphor-PPAR-α, PPAR-γ and phosphor-PPAR-γ. MAPK inhibitors SP600125, U0126 and SB203580 (MAPK inhibitors) were obtained from Cell Signaling Technology (Danvers, MA, USA). Goat anti-mouse IgG HRP-conjugated antibody was purchased from Southern Biotech (Birmingham, AL, USA). Goat anti-rabbit IgG HRP-conjugated antibody was purchased from Invitrogen (Carlsbad, CA, USA). The pNF-κB-Luc, pAP-1-Luc reporter vectors and the pRL-TK internal control vector were purchased from Promega (Madison, WI, USA). Enhanced chemiluminescence (ECL) substrate was bought from Thermo Scientific (Waltham, MA, USA).

### 4.2. Isolation of Rice Bran Oil by Supercritical Carbon Dioxide Extraction 

The bran was dried using a commercial dryer (STERIS, Worcester, MA, USA) for 3 min at 110 °C in a vacuum sealed plastic pouch and was stored at −20 °C for further use. Each rice bran sample of 100 g was accurately weighed through analytical balance and was loaded into a 200 mL high-pressure vessel equipped with a water jacket (HanYang Sci., Seoul, Korea). The CO_2_ flow rate was routinely kept constant at 2.5 L/min. In every experimental design, the extracted oil was weighed with an analytical balance at set time intervals. Supercritical CO_2_ extraction was carried out using HA 221-50-06-C (Huaan Company Ltd., Nantong, China). Pure CO_2_ was applied by using a high pressure pump 2TB-50 (Huaan Company Ltd., Nantong, China). According to Yoon [29], the extraction temperature was set at 60 °C, and the extraction pressure was set at 27.6 MPa for 60 min. The SC-CO_2_ extraction experiments at each specific combination of pressure and temperature were performed in triplicate.

### 4.3. High Performance Liquid Chromatography (HPLC)

The rice bran oil extracts were dissolved in methanol, were further filtered with a 0.45 μm filter and were analyzed by HPLC. δ-Tocotrienol standard sample was purchased from Sigma (Sigma Co., Ltd., St. Louis, MO, USA). HPLC analysis was performed on a Shimadzu Prominence series apparatus with a fluorescence detector (Prominence RF-20A/Axs, Shimadzu Co., Ltd., Kyoto, Japan). The excitation wavelength was 296 nm and the emission wavelength was 325 nm, which were operated on fluorescence detector. The Hypersil Gold PFP column (250 mm × 4.6 mm i.d., 5 μm, ThermoFisher Scientific, Waltham, MA, USA) was used as an analytical column. The eluents were methanol/H_2_O (85:15, *vol*/*vol*) at a flow rate of 0.8 mL/min. All the results were recorded, and the peaks were integrated by the chromatography software Labsolution LC (Shimadzu, Kyoto, Japan). δ-Tocotrienol was purified by Shim-pack PREP-ODS (20.0 mm × 250 mm, 15 µm, Shimadzu, Kyoto, Japan). The eluents include acetonitrile, tetrahydrofuran, methanol, 1% ammonium acetate (684:220:68:28) and the flow rate was 8 mL/min. The δ-tocotrienol standard that was purchased from Chromadex, Inc. (Irvine, CA, USA) was used to determine the absorption peak of δ-tocotrienol.

### 4.4. Cell Culture 

RAW264.7 is a mouse monocyte-macrophage cell line and was purchased from the Institute of Cell Biology, Chinese Academy of Science, Shanghai, China. RAW264.7 cells were cultured in RPMI 1640 medium that was supplemented with 10% FBS (Gibco-BRL, Carlsbad, CA, USA) at 37 °C in a humidified incubator with 5% CO_2_ atmosphere. The experiment details were described in our recent publication [32]. δ-Tocotrienol that was extracted from rice bran was dissolved in ethanol.

### 4.5. Cell Viability Assay 

Cell viability was evaluated by the CellTiter 96 Aqueous One Solution Proliferation Assay Kit (Promega). The treated cells were incubated for 24 h, and then growth medium was replaced by a solution of 100 μL of fresh growth medium and 20 μL of MTS. The plate was incubated for another 2 h at 37 °C and the absorbance was measured at 490 nm. The percentage of cell viability relative to ethanol (solvent control) was calculated. 

### 4.6. Observation of Morphological Changes 

The morphological change of murine macrophages RAW264.7 is considered one of the remarkable characteristics of the toxicological effect. To determine whether δ-tocotrienol was toxicant to RAW264.7 cells, an optical microscopy (DM2500, Leica, Solms, Germany) was used to detect the toxicity of δ-tocotrienol and was compared with the control group. After 72 h of δ-tocotrienol (0 μM, 5 μM, 10 μM, 20 μM, 40 μM, 80 μM) treatment on RAW264.7 cells, the morphological changes, including cell floating and shrinkage and nucleic blebbing, were observed.

### 4.7. RNA Isolation and RT-qPCR 

Total RNA from murine macrophages RAW 264.7 cells was extracted by the Trizol reagent kit (Transgen, Beijing, China) and was then reverse transcribed by using high-Capacity cDNA Reverse Transcription Kits (Applied Biosystems, Foster City, CA, USA). Consistent with Liu et al. [28] and Guo et al. [32], the relative mRNA expression levels of pro-inflammatory factors and iNOS were analyzed by real-time quantitative PCR (RT-qPCR). Cells were treated with δ-tocotrienol (5 μM, 10 μM, 20 μM) for 2 h followed by adding LPS (1 µg/mL) for 6 h. According to the manufacturer’s protocol, 1 μg total RNA extracted from RAW264.7 cells was used in reverse transcription reaction with the One Step RT-PCR kit (Gibco-BRL). PCR amplifications were carried out for 32 cycles and each cycle consisted of a denaturing step for 3 min at 94 °C, a further denaturing at 94 °C for 30 s, an annealing step for 30 s at 60 °C and a polymerization step for 1 min at 72 °C. The PCR primer was according to Guo’s publication [32].

### 4.8. Extraction of Nuclear and Cytosolic Proteins 

RAW264.7 cells were cultured in a 100 mm dish at a density of 1 × 10^6^ cells/mL for 24 h. After incubation, the cells were treated with various concentrations of δ-tocotrienol (0.5 μM, 10 μM and 20 μM) for 2 h and 1 μg/mL of LPS was then added for 60 min. Total protein from the cells was extracted with radio immunoprecipitation assay (RIPA) buffer (2 mM PMSF, 2 mM EDTA and 2 mM orthovanadate, 1% Triton X-100, 0.5% SDS, 0.1% deoxycholate) that was supplemented with a cocktail of protease and phosphatase inhibitors. RAW264.7 cells were harvested, and nuclear and cytosolic fractions were prepared using a Nuclear Extraction Kit (Sigma-Aldrich, St. Louis, MO, USA) according to the manufacturer’s instructions.

### 4.9. Western Blot Analysis 

RAW264.7 cells (1 × 10^6^) were lysed in Tris buffered saline Tween (TBST) buffer (50 mM Tris, pH 7.6, 150 mM NaCl, and 0.05% Tween-20) and protease and phosphates inhibitors were added. Nuclear and cytoplasmic extraction kits were used to collect nuclear and cytoplasmic proteins. Equal amounts of nuclear, cytoplasm or whole cell extracts were separated by 13% SDS-PAGE, were transferred to nitrocellulose filters and blocked for 1 h and were then incubated with the corresponding antibodies to β-Actin (1:1000), IL-1β (1:1000), TNF-α (1:1000), IL-6 (1:1000), iNOS (1:500), IκB-α (1:500), c-Jun (1:1000), Phospho-c-Jun (1:500), phospho-ERK1/2 (1:1000), phospho-JNK (1:500) and phospho-p38 (1:500) at 4 °C overnight. The membrane was washed three times with Tris-bufffered saline, containing 0.05% Tween 20 (TBST) for 10 min and was incubated with anti-rabbit or anti-mouse IgG-horseradish peroxidase (1:5000, Pierce, Waltham, MA, USA) at room temperature for 1 h. The protein bands were visualized using an ECL system following the manufacturer’s instructions.

### 4.10. Luciferase Reporter Assay 

RAW264.7 cells were seeded in 24 well plates (Falcon Plastics, Oxnard, CA, USA) and were then transiently transfected at 80% confluency, with either 1.0 μg NF-κB-luc or AP-1-luc reporter plasmid DNA along with 0.5 μg SV40-β-galactosidase expression construct DNA (pSV β-gal) as an internal control, and 1.0 μg of the empty vector phRL-TK, using Lipofectamine 2000 (Invitrogen Life Technologies, Carlsbad, CA, USA) following the manufacturer’s protocol. 

### 4.11. Statistical Analysis

For statistical analysis, SPSS17.0 software (Chicago, IL, USA) was employed. One-way ANOVA or student’s *t*-test was used for determining the statistically significant differences between the values of various experimental and control groups. Data was expressed as means ± SD, and a *p* value of 0.05 was considered statistically significant and of 0.01 was considered statistically very significant.
